# Comparison data of common and abundant terpenes at different grape development stages in Shiraz wine grapes

**DOI:** 10.1016/j.dib.2016.07.010

**Published:** 2016-07-14

**Authors:** Pangzhen Zhang, Sigfredo Fuentes, Tracey Siebert, Mark Krstic, Markus Herderich, Edward William R. Barlow, Kate Howell

**Affiliations:** a Faculty of Veterinary and Agricultural Sciences, University of Melbourne, Parkville, VIC 3010, Australia; b Australian Wine Research Institute, Urrbrae, SA 5064, Australia; c Australian Wine Research Institute, Mooroolbark, VIC 3138, Australia; d INRA, UMR1083 SPO, 2, Place Viala, F-34060 Montpellier, France

**Keywords:** Rotundone, Terpene, Sesquiterpene, Grape ripening

## Abstract

Terpenoids were extracted from grape vine bunches during plant development and analysed by GC-MSD. The grapevines analysed were from a commercial harvest of Vitis vinifera cv. Shiraz. The terpenoids were analysed from 4 weeks post flowering (wpf) to harvest in one season. The data are presented with the structure of the compound and aroma profile and semi-quantified. The sub-class of sesquiterpenes was given special attention, and this data set describes the first analysis of these compounds during ripening of this important economic crop. Sesquiterpenes may have a hitherto described contribution to wine aroma. This data set may provide insight into biosynthetic pathways and aroma chemistry. Interpretation of our data and further discussion can be found in “Terpene evolution during the development of Vitis vinifera L. cv. Shiraz grapes” (Zhang et al., 2016) [1].

## **Specifications table**

**Table:** 

Subject area	*Chemistry, Biology*
More specific subject area	*Aroma chemistry of wine grapes*
Type of data	*Table*
How data was acquired	*Gas chromatography- mass spectrometry.* An Agilent Technologies 6890 gas chromatograph (GC; Agilent Technologies, Santa Clara, CA) was equipped with a Gerstel MPS2 multipurpose sampler and coupled to an Agilent 5973 mass selective detector (MSD).
Data format	*Analyzed*
Experimental factors	Grape samples were homogenized and the volatile fractions directly sampled using SPME.
Experimental features	The physiological stages of grapevine ripening were comprehensively sampled, from weeks post flowering (wpf) until physiological ripening. Grape samples were homogenized, extracted and the sesquiterpene fraction qualified and quantified to compare the concentration and accumulation over time.
Data source location	The Old Block, Mount Langi Ghiran 37.31°S, 143.15°E, Victoria, Australia
Data accessibility	*Data is with this article*

## **Value of the data**

• This data is a comprehensive list of terpenoids from bunches during ripening of wine grapes and is presented by calculating the total peak area of the compound with the total terpenoid peak area. The data is also semi-quantified by presenting as µg α-copaene equivalents/ mean berry weight in kg. The structure of the compound and aroma descriptor (if known) is given.
• Comparison with other vineyard studies to gain insight into the cultivar-dependent synthesis of these compounds is valuable. Understanding the importance of weather and climate, cultural practices and maximizing aroma in wine could be areas of further investigation.
• Absolute quantification by synthesis of the deuterated analogs, importance to aroma of wine and understanding the biosynthetic pathways of terpenoids are possible areas of collaboration for future research.

## 1 Data

Terpenoids are important plant secondary compounds and wine aroma compounds. Terpenoids were analysed in grapevines (*Vitis vinifera cv. Shiraz*) during physiological ripening, from weeks post flowering (wpf) (Table 1).

## 2 Experimental design, materials and methods

The vineyard is located approximately 15.5 km east to the nearest Bureau of Meteorology (BOM) weather station (Ararat Prison Station, Vic, Australian BOM Station No. 089085). The long-term mean January temperature (MJT) and annual average rainfall recorded at this weather station by February 2015 is 19.1 °C and 584.2 mm, respectively. Therefore, the viticulture region is classified as a cool climate wine region [2]. The MJT and total rainfall from October to harvest for the studied season (2013–14) was 20.0 °C and 124.1 mm. The *Vitis vinifera*, cv. Shiraz vineyard was planted in 1968 with on its own roots, 3.0 m between rows and 1.8 m between vines, with rows oriented northeast to southwest. Vertical shoot positioned (VSP) trellis system was used. Grapevines were irrigated when required at a rate of 5.76 L/(hr vine) using a dripping irrigation system along vineyard rows with a dripper spacing of 0.5 m. The total irrigation volume for the studied season (2013–14 October to harvest) was 84.3 mm. No significant pest or disease pressure was observed during the experiment.

Terpenoid analysis was conducted for grape samples based on a published protocol [3] with the following modifications. An Agilent Technologies 6890 gas chromatograph (GC; Agilent Technologies, Santa Clara, CA) equipped with an Agilent 5973 mass selective detector (MSD) was used. A Gerstel MPS2 multipurpose sampler was used to control head-space solid phase microextraction (HS-SPME) and injection. The instruments were controlled using Agilent G1701EA MSD ChemStation software and Gerstel Maestro software (version 1.4.20.0). The GC was fitted with a J&W DB-5ms capillary column measuring approximately 30 m ×0.25 mm, 0.25 μm film df. Helium (ultrahigh purity, BOC, Adelaide, SA, Australia) was used as carrier gas in constant flow mode (1.0 ml/min). The GC inlet was fitted with a resilanised borosilicate glass SPME inlet liner (Supelco, 6.5 mm o.d., 0.75 mm i.d., 78.5 mm long) held at 220 °C.

The SPME fibre was desorbed in the pulsed splitless mode and the splitter, at 50:1, was opened after 30 s. The fibre was allowed to bake in the inlet for 10 min. The oven was started at 50 °C, held at this temperature for 1 min, then increased to 230 °C at 3 °C/min, and increased to 280 °C at 20 °C/min and held at 280 °C for 5 min. The temperatures of the MS source and quadruple were set at 230 °C and 150 °C, respectively. The MS transfer line was held at 250 °C. Simultaneous selective ion monitoring (SIM) and scanning modes were used to record electron ionization mass spectrometric data in the range of 35–280 *m*/*z* with ionization voltage of 70 eV.

100 g of representative de-stemmed grapes were homogenised using a hand-held blender. 5 g of homogenised sample was weighted into a HS-SPME vial (Agilent Technologies, 20 ml), mixed with 500 μL α-copaene (200.64 μg/L in ethanol) as internal standard, and shaken for 24 h at 22 °C. 2 ml saturated brine were then added to the samples before subjected to SPME-GC–MS analysis. The vial and its contents were heated to 45 °C. The polydimethylsiloxane/divinylbenzene (PDMS/DVB, Agilent) 65μm SPME fibre was exposed to the headspace for 60 min with agitation. Sesquiterpenes were identified by comparing the mass spectra and retention indices with the terpenoids library in MassFinder (version 4.1, Dr. Hochmuth Scientific Consulting, Hamburg, Germany). All compounds except α-guaiene were semi-quantified as α-copaene equivalents, expressed as relative areas×100. α-guaiene was determined by SIM with α-copaene as internal standard; the ions monitored were: *m*/*z* 105, 133, 147, 161, and 204; dwell time 25 ms each. The target ions used were *m*/*z* 147 for α-guaiene and 161 for α-copaene with ions 105, 133, and 204 *m*/*z* used as qualifiers. Data were analysed using Agilent G1701DA MSD ChemStation software. α-Guaiene was expressed as the ratio of *m*/*z* 147:*m*/*z* 161 multiplied by the concentration of α-copaene internal standard. The assay precision was validated by a series of standard additions of internal standard as described previously [3]. Blank SPME runs and blank internal standards were checked regularly.

Comparison of terpenoid profiles at different berry developmental stages were analysed by discriminant analysis using SPSS v.21 (SPSS Inc., Chicago, IL. USA).

## Figures and Tables

**Table 1:** Comparison of common and abundant terpenes at different grape development stages in the 2013-14 growing season^a^.

GC Peak number	Compound number	Terpenes compounds and classes		Chemical structures^b^	Odour quality^c^	Concentration at different weeks post-flowering (wpf) (µg α-copaene equivalents/ mean berry weight in kg) Percentage of individual terpenoids to total terpenoids in terms of GC peak area						
						wpf 4	wpf 6	wpf 9	wpf 11	wpf 13	wpf 15	wpf 17
						E-L 31	E-L 32	EL 34-35	E-L 35-36	E-L 36	E-L 37	E-L 38
		**Monoterpenes (Monoterpenoids)**										
1	10	Limonene			lemon, orange	6.25±0.76	ND^d^	ND	ND	ND	ND	ND
						1.12%						
2	12	1,8-Cineole			mint, sweet	7.94±0.66**a**	2.21±1.6**b**	1.34±0.14**b**	1.21±0.61**b**	1.73±0.31**b**	2.37±0.92**b**	1.87±1.27**b**
						1.43%	1.34%	3.20%	4.73%	6.03%	7.72%	4.21%
3	9	Geraniol			rose, geranium	9.31±6.33**a**	5.57±3.16**ab**	1.06±0.14**b**	2.17±0.72**b**	5.73±1.66**ab**	6.48±0.42**ab**	6.36±0.59**ab**
						1.67%	3.38%	2.53%	8.49%	19.99%	21.12%	14.30
16	7	Geranyl acetone^e^			magnolia, green	27.18±5.86**a**	22.39±7.31**a**	5.16±1.76**b**	1.13±0.54**b**	1.30±0.31**b**	1.62±0.25**b**	1.77±0.15**b**
						4.89%	13.58%	12.33%	4.45%	4.54%	5.28%	3.99%
27	15	Citronellol			rose	1.47±0.47**a**	2.88±1.33**b**	0.79±0.44**a**	ND	ND	ND	ND
						0.26%	1.75%	1.89%				
		**Sesquiterpenes**										
6	19	Clovene			NA^f^	0.55±0.09**a**	ND	ND	0.52±0.19**a**	0.26±0.06**b**	0.25±0.06**b**	0.54±0.16**a**
									2.05%	0.91%	0.81%	1.22%
						0.10%						
7	51	α-Ylangene			NA	ND	ND	ND	ND	ND	1.40±0.44^g^**a**	4.16±0.51**b**
											4.56%	9.36%
10	53	β-Bourbonene			herb	ND	ND	ND	ND	ND	ND	0.77±0.27
												1.74%
		**Sesquiterpenes**										
11	18	(E)- β-Caryophyllene			wood, spice	166.65±24.10**a**	27.12±4.52**b**	4.72±0.93**c**	ND	ND	ND	ND
						29.95%	16.45%	11.30%				
12	54	β-Copaene			NA	ND	ND	ND	ND	ND	ND	1.19±0.09
												2.67%
13	22	α-Guaiene^h^			wood, balsamic	0.09±0.03**ab**	0.26±0.04**c**	0.07±0.03**ab**	0.03±0.01**a**	0.02±0.01**a**	0.01±0.01**a**	0.11±0.08**b**
						0.02%	0.16%	0.16%	0.10%	0.06%	0.05%	0.24%
14	30	Guaia-6,9-diene			NA	ND	ND	ND	ND	ND	ND	1.26±0.20
												2.84%
15	52	Selina-4(15),6-diene			NA	ND	ND	ND	ND	ND	ND	0.44±0.23
												0.99%
17	20	α-Humulene			wood	188.50±33.58**a**	18.12±5.48**b**	1.78±0.42**b**	1.02±0.31**b**	0.32±0.21**b**	0.86±0.19^f^**b**	0.88±0.55**b**
						33.88%	10.99%	4.26%	3.98%	1.13%	2.79%	1.97%
18	40	γ-Muurolene			herb, wood, spice	3.67±1.20**a**	0.86±0.23**b**	ND	ND	ND	ND	ND
						0.66%	0.52%					
		**Sesquiterpenes**										
20	27	δ-Selinene			NA	ND	ND	ND	ND	ND	ND	1.20±0.28
												2.70%
21	46	*epi*-Zonarene			NA	ND	ND	ND	ND	ND	0.14^i^	3.15±0.33
											0.47%	7.09%
22	38	α-Muurolene			wood	6.44±0.74**a**	3.29±0.74**b**	ND	ND	ND	ND	ND
						1.16%	2.00%					
23	34	γ-Cadinene			wood	ND	ND	ND	ND	ND	ND	1.89±0.22
												4.26%
24	39	δ-Cadinene			thyme, medicine, wood	40.28±5.44**a**	11.08±0.83**b**	1.83±0.47**c**	0.95±0.16**c**	0.95±0.92**c**	0.33±0.02**c**	1.39±0.35**c**
						7.24%	6.72%	4.37%	3.71%	3.31%	1.09%	3.12%
25	48/49	*Cis/trans*-Calamenene			herb, spice	ND	2.47±0.13**a**	0.18±0.15**c**	0.10±0.04**c**	0.10±0.05**c**	0.14±0.03^g^**c**	0.77±0.01**b**
							1.50%	0.44%	0.41%	0.34%	0.45%	1.72%
26	47	Zonarene			NA	29.52±2.54**a**	2.19±0.52**b**	0.16±0.18^g^**b**	0.05±0.02^f^**b**	ND	ND	ND
						5.31%	1.33%	0.37%	0.19%			
		**Sesquiterpenes**										
28	28	7-*epi*-α-Selinene			NA	ND	ND	ND	ND	ND	ND	0.73±0.26
												1.65%
29	36	ω-Cadinene			NA	6.24±0.84**a**	1.79±0.29**b**	ND	ND	ND	ND	ND
						1.12%	1.09%					
30	35	α-Cadinene			NA	ND	ND	ND	ND	ND	ND	0.74±0.12
												1.66%
31	42	α-Calacorene			wood	4.44±0.51**a**	3.01±0.18**b**	0.26±0.18**c**	0.11±0.03**c**	ND	ND	0.49±0.33**c**
						0.80%	1.83%	0.62%	0.42%			
												1.10%
32	57	*Epi*-Cubenol			NA	5.47±0.19	ND	ND	ND	ND	ND	ND
						0.98%						
33	NA	Cubenol			spice, herb, green tea	5.43±0.63	ND	ND	ND	ND	ND	ND
						0.98%						
		**Norisoprenoids**										
4	60	Theaspirane isomer A			fruits, peach, honey	21.14±1.78**a**	32.16±4.57**b**	14.30±0.62**c**	10.90±2.23**c**	12.08±1.09**c**	13.64±0.76**c**	11.49±2.71**c**
						3.80%	19.51%	34.19%	42.74%	42.15%	44.48%	25.83%
5	60	Theaspirane isomer B			camphor, mint, wood, eucalyptus	8.36±1.23**a**	13.19±2.44**b**	5.39±0.08**c**	3.31±0.88**d**	2.87±0.31**d**	2.58±0.08**d**	1.74±0.28**d**
						1.50%	8.00%	12.90%	12.97%	10.02%	8.43%	3.91%
9	62	(E)-β-Damascenone			apple. rose, honey	4.61±0.86**a**	5.01±1.07**a**	2.10±0.28**b**	1.97±0.50**b**	1.36±0.56**b**	ND	ND
						0.83%	3.04%	5.02%	7.73%	4.75%		
19	61	β-Ionone			seaweed, violet, flower, raspberry	12.85±2.16**a**	11.23±1.46**b**	2.73±0.15**c**	2.07±0.31**c**	1.94±0.22**c**	1.64±0.22**c**	1.52±0.73**c**
						2.31%	6.81%	6.54%	8.11%	6.78%	5.35%	3.42%
	**Total monoterpenoids**					52.15±10.74**a**	33.06±11.62**b**	8.34±1.98**c**	4.51±0.96**c**	8.76±2.05**c**	10.46±1.25**c**	10.01±1.75**c**
						9.37%	20.06%	19.95%	17.67%	30.56%	34.12%	22.50%
	**Total norisoprenoids**					46.95±4.76**a**	61.60±9.44**b**	24.53±0.84**c**	18.25±3.89**c**	18.26±2.05**c**	17.87±0.96**c**	14.75±3.60**c**
						8.44%	37.37%	58.65%	71.54%	63.70%	58.26%	33.16%
	**Total sesquiterpenes**					457.27±66.87**a**	70.19±10.62**b**	8.95±1.42**c**	2.75±0.68**c**	1.65±1.12**c**	2.34±1.56**c**	19.72±2.71**c**
						82.19%	42.58%	21.40%	10.79%	5.75%	7.62%	44.34%
	**Total terpenoids**					556.37±54.62**a**	164.84±30.14**b**	41.81±1.15**c**	25.51±5.00**c**	28.67±3.80**c**	30.67±1.46**c**	44.47±6.72**c**
	**Total volatile compounds**					2297.53±495.00**a**	1564.11±517.35**b**	592.44±77.34**c**	511.38±58.63**c**	674.18±69.08**c**	859.16±60.13**c**	750.77±87.62**c**
	**Percentage of terpenoids to total volatile compounds**					24.22%	10.54%	7.06%	4.99%	4.25%	3.57%	5.92%

a Different letters in the column represent significantly different means ± standard error (*p*<0.05).

b The enantiomeric purity of most chiral compounds is unknown, and so here a single enantiomer was chosen to represent each compound.

c Odour of the compound taken from Flavornet by Terry Acre and Heinrich Arn, http://www.flavornet.org © Datu Inc., 2014, and Leffingwell & Associates, http://www.leffingwell.com © Leffingwell & Associates, 2014.

d Not detected.

e Geranyl acetone is a monoterpenoid, not a monoterpene.

f Not available.

g The compound was detected in 2 replicates only.

h α-Guaiene was expressed as the ratio of m/z 147: m/z 161 multiplied by the concentration of internal standard, α-Guaiene was expressed as the ratio of m/z 147: m/z 161 multipliedy by the concentration of internal standard, α-copaene.

i The compound was detected only in 1 replicate only.

## References

[1] Zhang P., Fuentes S., Siebert T., Krstic M., Herderich M., Barlow E.W.R., Howell K. (2016). Terpene evolution during the development of *Vitis vinifera* L. cv. Shiraz grapes. Food Chem..

[2] Gladstones J.S., Dry P.R., Coombe B.G. (2004). Climate and Australian viticulture.

[3] Parker M., Pollnitz A.P., Cozzolino D., Francis I.L., Herderich M.J. (2007). Identification and quantification of a marker compound for ‘Pepper׳ aroma and flavor in shiraz grape berries by combination of chemometrics and gas chromatography−mass spectrometry. J. Agric. Food Chem..

