# Supplementary material for: Comparison data of common and abundant terpenes at different grape development stages in Shiraz wine grapes
Source: Data Brief. 2016 Jul 14;8:1127–36. doi: 10.1016/j.dib.2016.07.010 (PMC4983143; doi:10.1016/j.dib.2016.07.010)
Supplement: Supplementary file 1 — Supplementary material [file mmc1.docx]

Conflict of interest statement

The authors have the following interests: The field work in this study was supported by a commercial company (Rathbone Wine Group) and the research body of the Australian Wine Research Institute. Co-authors Mark Krstic, Tracey Siebert and Markus Herderich are employed by Australian Wine Research Institute. There are no patents, products in development or marketed products to declare.
